# Outcomes in dialysis versus conservative care for older patients: A prospective cohort analysis of stage 5 Chronic Kidney Disease

**DOI:** 10.1371/journal.pone.0206469

**Published:** 2018-10-26

**Authors:** Maharajan Raman, Rachel J. Middleton, Philip A. Kalra, Darren Green

**Affiliations:** 1 Vascular Research Group, Salford Royal NHS Foundation Trust, Salford, United Kingdom; 2 Faculty of Biology, Medicine and Health, University of Manchester, Manchester, United Kingdom; University of Wisconsin, UNITED STATES

## Abstract

**Background:**

The benefits of dialysis in older people with ESKD are not clear. We prospectively evaluated whether dialysis has survival advantage compared to conservative care (CC) in older people who were medically suitable for dialysis therapy.

**Methods:**

This was a prospective observational study of CKD patients aged ≥75 years when eGFR first reached ≤15ml/min/1.73m^2^. Hazard ratios (HR) for death were compared between patients who chose dialysis versus conservative care (CC) from when first seen in pre-dialysis clinic (eGFR ≤15ml/min/1.73m^2^), and when initiation of dialysis was first considered (eGFR ≤10ml/min/1.73m^2^). Patients with co-morbidities likely to significantly reduce life expectancy such as advanced heart failure, advanced dementia, and malignancy, were excluded.

**Results:**

There were 204 patients (123 dialysis, 81 CC). 115 went on to record eGFR of ≤10ml/min/1.73m^2^ (73 dialysis, 42 CC). The median survival from eGFR first ≤15ml/min/1.73m^2^ for the dialysis and CC groups were 42 (33–50) months and 31 (21–41) months. The adjusted hazard ratio (HR) for death in the dialysis group compared to CC was 0.61 (0.41–0.61, p = 0.01). The median survival from eGFR first ≤10ml/min/1.73m^2^ for dialysis and CC group were 36 (25–47) months and 12 (0–5) months. The adjusted HR for death in the dialysis group compared to CC was 0.36 (0.21–0.62, p <0.001).

**Conclusion:**

Dialysis confers a survival benefit in older patients medically suitable for dialysis. This study is novel in being both prospective and in excluding patients with co-morbidities which may limit suitability for dialysis and life expectancy. A future focus on quality of life is needed to establish the true benefits of dialysis in older people.

## Introduction

Older people with CKD have an accumulation of co-morbidities and lower life expectancy compared to younger patients. This contributes to the current lack of clarity around whether dialysis is beneficial in older people. One year mortality for prevalent RRT patients aged 75 to 79 years is high at 200 per 1000 patient years, and higher still for the age group > 85 years at 371 per 1000 patient years [1]. This compares with 75 per 1000 patient years in the age group 60 to 65 years, and 127 per 100 patient years in patients aged >85 years without CKD.

It has been suggested that older age has been used covertly to ration dialysis [2]. However, according to the United Kingdom (UK) Renal Registry, the incident rate of commencing renal replacement therapy (RRT) is actually highest among the age group 75 to 79 years, at approximately 500 per million population in the UK.

The aim of this study was to establish whether dialysis confers a survival benefit compared to conservative therapy for older patients from two time points: a) when eGFR first falls below 15mL/min/1.73m^2^, and b) when eGFR first falls below 10mL/min/1.73m^2^. These time points were chosen as they respectively reflect two important time points on the patient journey through outpatient renal services. The first being the point at which pre-dialysis counselling and modality discussions typically begin, and the second is point at which dialysis itself would typically begin or be considered to be appropriate.

We also selected only patients who first developed stage 5 CKD in an outpatient setting, omitting AKI requiring dialysis and “crash-landers” who presented to hospital with stage 5 CKD as an acute medical emergency.

Collectively, these criteria produce an analysis which could be used to provide patients with information about their future care and prognosis, specifically in the context of a pre-dialysis clinic or home visit, the places where decisions about whether or how to dialyse are typically made, such that they may be able to make a clear informed decision.

As secondary analyses, we compared any apparent survival benefit conferred by dialysis with the number of extra in-patient and out-patient hospital days faced by dialysis patients. We also observed whether any demographic factors significantly influenced the decision to dialyse or choose conservative care (e.g. being widowed or living alone), and whether there is any survival difference between those who selected haemodialysis and peritoneal dialysis.

## Materials and methods

This was a sub-study of the Salford Kidney Study; a single centre prospectively collected observational study of outcomes in Chronic Kidney Disease in the United Kingdom. Patients who are referred to the Nephrology Secondary Care outpatient clinic at Salford Royal NHS Foundation Trust, or admitted to the Nephrology inpatient ward were approached for inclusion in the study, and were enrolled if written informed consent was gained. Patients underwent annual review including detailed clinical phenotyping and event reporting. Phenotype and outcome data were collected from patient self-reporting, Hospital Electronic Patient Records, primary care records, and mortality data from the Office for National Statistics. The study complies with the declaration of Helsinki and local ethical approval was obtained from the South Manchester Ethics Committee, UK (current REC reference 15/NW/0818).

Patients were retrospectively selected for this sub-group analysis who first recorded an outpatient eGFR ≤15mL/min/1.73m^2^ when aged ≥75 years. Exclusion criteria retrospectively applied were: NYHA 3 or 4 heart failure, previous cardiac arrest, solid organ malignancy diagnosed in the 5 years before eGFR ≤15mL/min/1.73m^2^, a Karnofsky performance score <60, any dementia diagnosis, dialysis solely for acute kidney injury (AKI), end stage kidney disease (ESKD) presenting as an emergency hospital admission, and patients with a planned pre-emptive live donor transplant.

Baseline data were collected for age, gender, eGFR, co-morbidities, Karnofsky Performance Score, marital status, prior occupation, habitation, and co-habitation details. All future outpatient eGFR measurements were recorded. Patients were assigned to either a dialysis group or conservative care group depending on the initial modality choice made by the patient after a home visit or face to face discussion with a pre-dialysis specialist nurse. Patients were excluded from the final analysis if they died before making a modality decision. In the results section, “dialysis patients” refers to patients who chose dialysis over conservative care rather than specifically those patients who began maintenance dialysis.

Follow up was from the date of first outpatient eGFR measurement of ≤15mL/min/1.73m^2^ in the first analysis, and from date of first outpatient eGFR measurement of ≤10mL/min/1.73m^2^ in the second analysis. In both cases, follow up was until death or 30^th^ April 2015. Date and cause of death data were obtained from the Office for National Statistics via the Health and Social Care Information Centre. We also compared survival in the period between eGFR 15 and eGFR 10mL/min/1.73m^2^ to determine if survival was comparable between the two groups in what would be the “pre-dialysis” period. This latter analysis was to determine if there was any signal of selection bias between groups i.e. whether the dialysis group had improved survival compared to the CC group even before initiation of renal replacement therapy. We also compared rate of change of eGFR between groups during this period.

Data were collected during follow up for inpatient and outpatient hospital days. Outpatient days included routine haemodialysis sessions, drop in visits, outpatient imaging, and outpatient clinic attendances. Inpatient days included day case surgery and procedures, and emergency department visits, as well as ward based inpatient stays. Data were also collected for the number of renal related invasive procedures. These included siting of temporary and tunnelled haemodialysis catheters, removal of tunnelled catheters, arteriovenous fistula formation, open or percutaneous insertion of peritoneal dialysis catheters, renal biopsies, and any procedure necessary as a result of a complication of any of these.

Between group comparisons of baseline characteristics in dialysis versus conservative care patients were undertaken using chi square tests, un-paired t-tests and Mann Whitney U tests depending on the characteristics and distribution of each variable.

Survival analyses were performed comparing dialysis patients versus conservative care patients using a Cox proportional hazard model adjusted for any variable which statistically differed between groups on the between group comparisons described above and which may influence survival. Further analyses compared outcomes in the population sub-groups of those aged ≥85 years, and patients with prior atherosclerotic cardiovascular events. Within the dialysis group, we compared patients who chose haemodialysis versus those who chose peritoneal dialysis.

Survival analysis was then repeated using propensity score matching. Propensity scores were created using clinical co-variates (age, gender, eGFR, diabetes, smoking status, individual cardiovascular co-morbidities, Karnofsky Performance Score, marital and co-habitation status). These were inputted into a logistic regression model with “dialysis patients” as the dependent variable. Dialysis and conservative care patients were then manually matched 1:1 with a calliper width of 0.01. Un-matched patients were excluded. Logistic regression was then used to calculate odds ratios for mortality at 1, 3, and 5 years after eGFR first <15 mL/min/1.73m^2^, and 1, 3, and 5 years after eGFR first <10 mL/min/1.73m^2^ in the dialysis versus CC matched patients.

The number of inpatient and outpatient days and the number of invasive procedures are expressed as annualised figures and compared between groups.

## Results

There were 258 patients in the final study group of patients who were aged ≥75 years at the point of first outpatient eGFR ≤15mL/min/1.73m^2^. 54 patients had not made any decision regarding dialysis versus conservative care during the follow up period and either died before any decision was made or remained undecided at the end of follow up. These were excluded.

**Fig 1:**
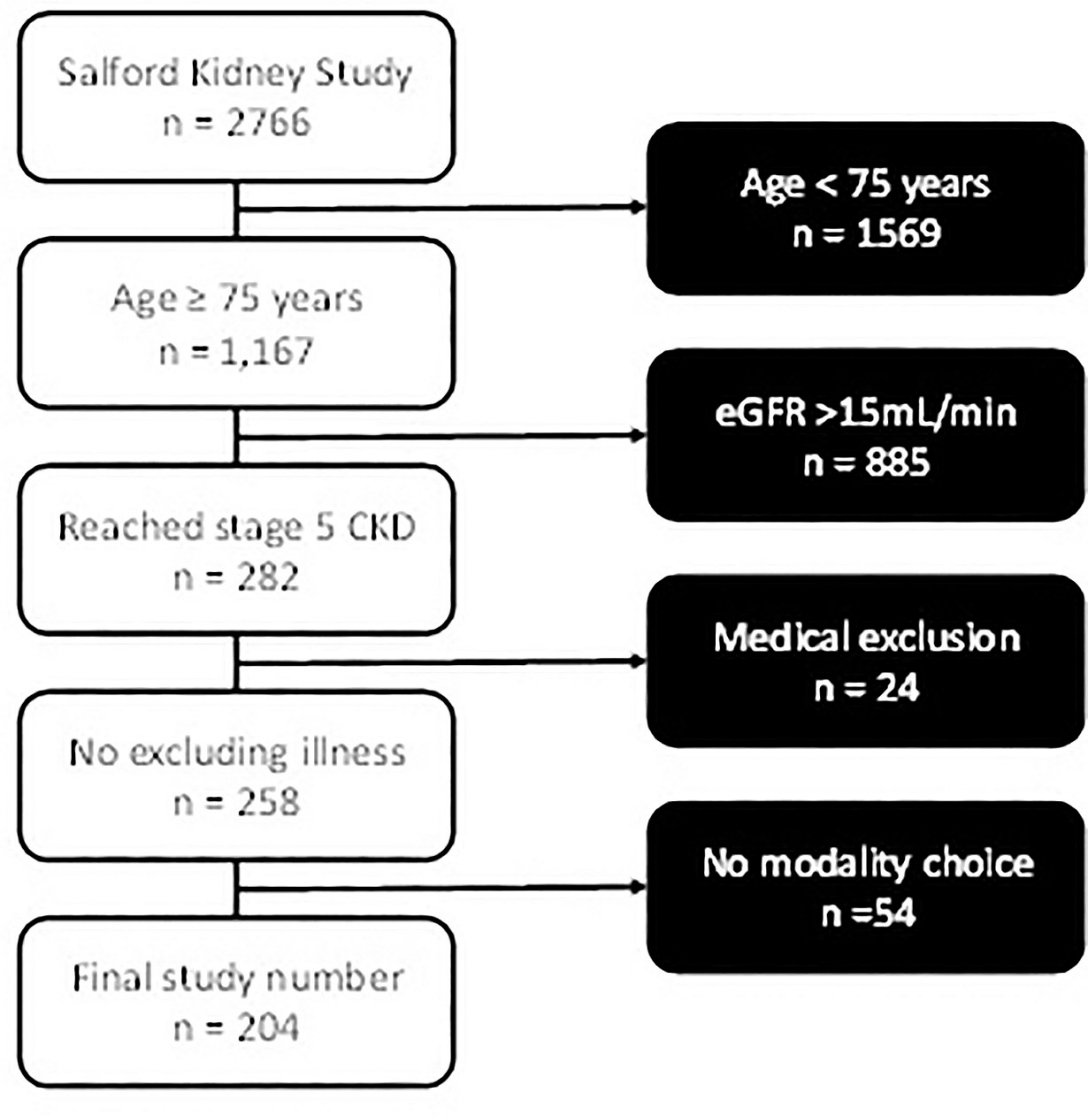
Reasons for exclusion from the study.

Of the final 204 patients, 123-chose dialysis (60%), and 81 elected for conservative care (40%). A flowchart outlining the exclusion of patients is shown in Fig 1. Patients who chose conservative care over dialysis were older (83.7 ± 4.2 years versus 78.9 ± 2.8 years, p <0.001), and more likely to live alone and have peripheral vascular disease (PVD). A full outline of baseline characteristics comparing the dialysis and conservative care groups is found in Table 1. Table 1 also compares baseline characteristics of dialysis patients who chose haemodialysis versus those who chose peritoneal dialysis. There was no difference seen between these groups.

**Table 1:** Between group comparisons at baseline of first outpatient eGFR ≤15 mL/min/1.73m^2^. Key: HD = haemodialysis; PD = peritoneal dialysis; CC = conservative care.

	Dialysis	CC	sig.	HD	PD	sig.
n	123	81		89	34	
Age (years)	78.9 ± 2.8	83.7 ± 4.2	<0.001	79.2 ± 2.9	78.2 ± 2.48	0.071
eGFR at baseline (mL/min/1.73m^2^)	12.9 ± 2.2	13.3 ± 2.2	0.196	12.9 ± 2.3	13.1 ± 1.9	0.864
Gender (% male)	66.7	55.6	0.109	64.0	73.5	0.318
CAD (%)	27.6	24.7	0.640	27.0	29.4	0.786
Heart failure (%)	48.8	45.7	0.664	50.6	44.1	0.523
COPD (%)	19.5	13.6	0.272	19.1	20.6	0.852
Diabetes (%)	39.8	29.6	0.137	36.0	50.0	0.155
PVD (%)	32.5	14.8	0.005	33.7	29.4	0.649
Amputation (%)	0.8	0.25	0.336	1.1	0.0	0.535
CVA (%)	25.2	18.5	0.264	27.0	20.6	0.466
Hemiplegia (%)	2.4	2.5	0.989	2.2	2.9	0.823
Systolic BP (mmHg)	143 ± 20	138 ± 19	0.288	143 ± 20	143 ± 33	0.994
Diastolic BP (mmHg)	68 ± 9	70 ± 11	0.551	70 ± 11	68 ± 16	0.483
Current or ex-smoker (%)	74.8	67.9	0.283	74.2	76.5	0.792
Alcohol intake (units / week)	3 ± 6	4 ± 8	0.575	4 ± 7	4 ± 6	0.866
Karnofsky score	88 ± 8	88 ± 9	0.822	88 ± 8	89 ± 8	0.602
Live alone (%)	21.1	39.5	0.004	23.6	14.7	0.280
Widowed (%)	23.6	37	0.187	22.5	26.5	0.673

**Abbreviations:** n, number; eGFR, estimation of glomerular filtration rate; CAD, coronary artery disease; COPD, chronic obstructive pulmonary disease; PVD, peripheral vascular disease; CVA, cerebrovascular accident; BP, blood pressure

Over a mean follow up from first eGFR ≤15 mL/min/1.73m^2^ of 35.1 ± 22.1 months, 52 of the dialysis patients (42.3%) started dialysis. For these patients, the median time to dialysis from first eGFR ≤15 mL/min/1.73m^2^ was 16.3 months (IQR, 9.2–27.5). No patients who chose conservative care switched to dialysis during follow up. Likewise, there were no transplants during follow up. In the dialysis group there were 72 deaths (58.5%), of which 44 (62% of deaths) were in patients who had not yet started dialysis. In the conservative care group there were 67 deaths (82.7%). There was no difference in the rate of change of eGFR between groups. The annualised mean rate of change of eGFR in dialysis patients was -2.7 ± 0.9 mL/min/1.73m^2^ per year, compared with -2.5 ± 1.3 mL/min/1.73m^2^ per year in the conservative care group.

**Fig 2:**
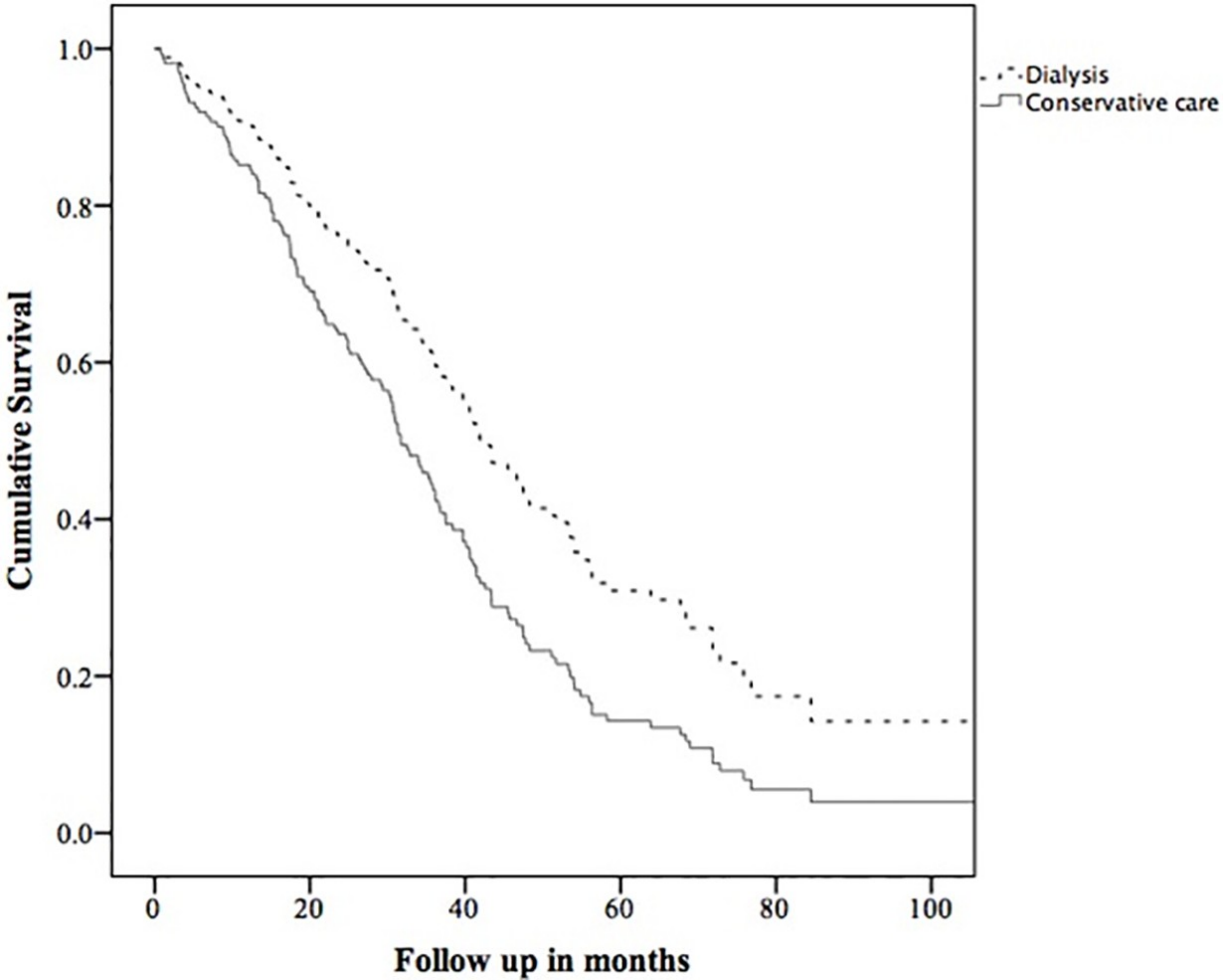
Survival curve comparing dialysis with conservative care from the date of first outpatient eGFR ≤15 mL/min/1.73m^2^. Adjusted for age, peripheral vascular disease, and living alone.

The survival analysis from first eGFR ≤15 mL/min/1.73m^2^ using a Cox proportional hazard model was adjusted for age, PVD and living alone as per Table 1. The adjusted hazard ratio for death in the dialysis group compared to the conservative care group was 0.61 (95% confidence intervals 0.41–0.91, p = 0.01). An adjusted survival curve comparing groups is shown in Fig 2. In the propensity score matching, 40 pairs were matched (mean propensity score in dialysis group = 0.452 ± 0.203, in CC group = 0.458 ± 0.207). In these matched pairs, one year after eGFR first <15 mL/min/1.73m^2^, the OR for mortality in the dialysis group compared to the conservative care group was 0.38 (0.09–1.60). At three years, the OR was 0.36 (0.12–1.06), and at 5 years was 0.70 (0.13–3.70). These are shown in Table 2.

**Table 2:** Comparison of survival between patients who chose dialysis versus those who chose conservative care, patients being matched 1:1 by propensity scores.

Mortality interval	Dialysis		Conservative		OR	95% CI		p
	n	(%)	n	(%)		Lower	Upper	
From eGFR first <15 mL/min/1.73m^2^								
1 year	3	(8)	7	(18)	0.38	0.09	1.60	0.19
3 year	10	(36)	17	(61)	0.36	0.12	1.06	0.06
5 year	14	(78)	15	(83)	0.70	0.13	3.70	0.67
From eGFR first <10 mL/min/1.73m^2^								
1 year	2	(10)	9	(45)	0.14	0.02	0.75	0.02
3 year	9	(53)	15	(88)	0.15	0.03	0.87	0.03
5 year	9	(82)	10	(91)	0.45	0.03	5.84	0.54

During follow up from first eGFR ≤15mL/min/1.73m^2^, the dialysis group spent statistically more inpatient and outpatient days at hospital compared to the conservative care group. The median annualised hospital days for dialysis patients was 23.4 (interquartile range [IQR], 10–85.9) compared with 10 (IQR, 5.2–24.2) for conservative care (p < 0.001). Dialysis patients also underwent more invasive renal procedures, and also more invasive non-renal procedures. Detailed comparisons of hospital activity are found in Table 3. For dialysis patients, Table 3 is further divided into those who chose haemodialysis versus those who chose peritoneal dialysis. Herein, patients who chose haemodialysis spent more time at hospital and underwent more procedures than peritoneal dialysis patients. In a Cox regression survival model comparing dialysis modalities, the HR for death in haemodialysis patients compared to peritoneal dialysis patients was 1.54 (0.85–2.76, p = 0.14).

**Table 3:** Comparisons of annualised number of hospital days and invasive procedures between dialysis and conservative care, and between haemodialysis and peritoneal dialysis from the baseline of first outpatient eGFR ≤15 mL/min/1.73m^2^ and first outpatient eGFR ≤10 mL/min/1.73m^2^. Key: HD = haemodialysis; PD = peritoneal dialysis. Numbers are expressed as median (interquartile range [IQR]).

	Dialysis	Conservative	sig.	HD	PD	sig.
From first outpatient eGFR ≤15 mL/min/1.73m^2^						
Outpatient days	14.1 (IQR, 8.3–55.7)	7.5 (IQR, 4–12.4))	<0.001	19.2 (IQR, 8.5–75.9)	11.9 (IQR, 7.9–15.3)	0.015
Inpatient days	2.2 (IQR, 0.7–14.7)	0.8 (IQR, 0.0–8.7)	0.005	4.3 (IQR, 0.9–17.3)	1.1 (IQR, 0.3–2.2)	0.003
Total hospital days	23.4 (IQR, 10–85.9)	10 (IQR, 5.2–24.2)	<0.001	38.5 (IQR, 10.8–96.9)	13.5 (IQR, 9.1–21.5)	0.002
From first outpatient eGFR ≤10 mL/min/1.73m^2^						
Outpatient days	34.3 (IQR, 9.9–92.8)	9.8 (IQR, 5.1–19.6)	<0.001	59.9 (IQR, 14.7–110.9)	12.2 (IQR, 3.7–21.3)	0.002
Inpatient days	4.1 (IQR, 1.1–20.2)	7.9 (IQR, 0.0–54.3)	0.729	8.2 (IQR, 1.6–25.1)	1.9 (IQR, 0.9–5.7)	0.153
Total hospital days	77.9 (IQR, 17.8–125.2)	20.7 (IQR, 7.8–79.8)	0.015	91.3 (IQR, 21.9–129.8)	17.9 (IQR, 9.2–48.9)	0.007

Of the 204 patients in the final study group, 115 survived to record an eGFR ≤10mL/min/1.73m^2^. Of these, 73 had chosen dialysis (63%), and 42 elected for conservative care (37%). The baseline characteristics at this point, including between group comparisons for dialysis versus conservative care and haemodialysis versus peritoneal dialysis, are found in Table 4. Again, conservative care patients were older and were more likely to have PVD.

**Table 4:** Between group comparisons at baseline of first outpatient eGFR ≤10 mL/min/1.73m^2^. Key: CC = conservative care group; dial = dialysis group; HD = haemodialysis; PD = peritoneal dialysis.

	Dial	CC	sig.	HD	PD	sig.
n	73	42		59	14	
Age (years)	79.8 ± 2.9	84.6 ±4.5	<0.001	79.8 ± 3.1	79.4 ± 2.1	0.678
eGFR at baseline (mL/min/1.73m^2^)	9.1 ± 1.2	8.9 ± 1.5	0.518	9 ± 1.3	9.7 ± 0.6	0.043
Gender (% male)	64.4	47.6	0.079	61	78.6	0.218
Coronary artery disease (%)	27.4	28.6	0.892	27.1	28.6	0.913
Heart failure (%)	38.4	42.9	0.635	42.4	21.4	0.147
COPD (%)	17.8	16.7	0.876	16.9	21.4	0.694
Diabetes (%)	32.9	28.6	0.632	35.6	21.4	0.310
PVD (%)	34.2	9.5	0.003	35.6	28.6	0.619
Amputation (%)	1.4	2.4	0.690	1.7	0.0	0.624
CVA (%)	26	16.7	0.248	25.4	28.6	0.809
Hemiplegia (%)	2.7	0.0	0.279	1.7	7.1	0.262
Systolic BP (mmHg)	146 ± 22	144 ± 20	0.638	146 ± 21	147 ± 26	0.855
Diastolic BP (mmHg)	71 ± 11	76 ± 11	0.057	71 ± 12	73 ± 10	0.717
Current or ex-smoker (%)	75.3	59.5	0.076	72.9	85.7	0.317
Alcohol intake (Units / week)	4 ± 6	4 ± 7	0.994	4 ± 6	6 ± 7	0.254
Karnofsky score	89 ± 9	90 ± 10	0.797	89 ± 9	91 ± 7	0.549
Live alone (%)	26	40.5	0.108	27.3	23.1	0.809
Widowed (%)	23.3	35.7	0.107	23.6	30.8	0.830

**Abbreviations:** n, number; eGFR, estimation of glomerular filtration rate; CAD, coronary artery disease; COPD, chronic obstructive pulmonary disease; PVD, peripheral vascular disease; CVA, cerebrovascular accident; BP, blood pressure

From first eGFR ≤10 mL/min/1.73m^2^, 47 of the dialysis patients (64%) started dialysis over a mean follow up of 26.9 ± 23.4 months. The median time to dialysis from first eGFR ≤10 mL/min/1.73m^2^ was 8.5 months (IQR, 3.2–15.1). In the dialysis group there were 45 deaths (62%) of which 19 (42% of deaths) were in patients who had not yet started dialysis. In the conservative care group there were 39 deaths (93% of conservative care patients who recorded an eGFR ≤10 mL/min/1.73m^2^).

**Fig 3:**
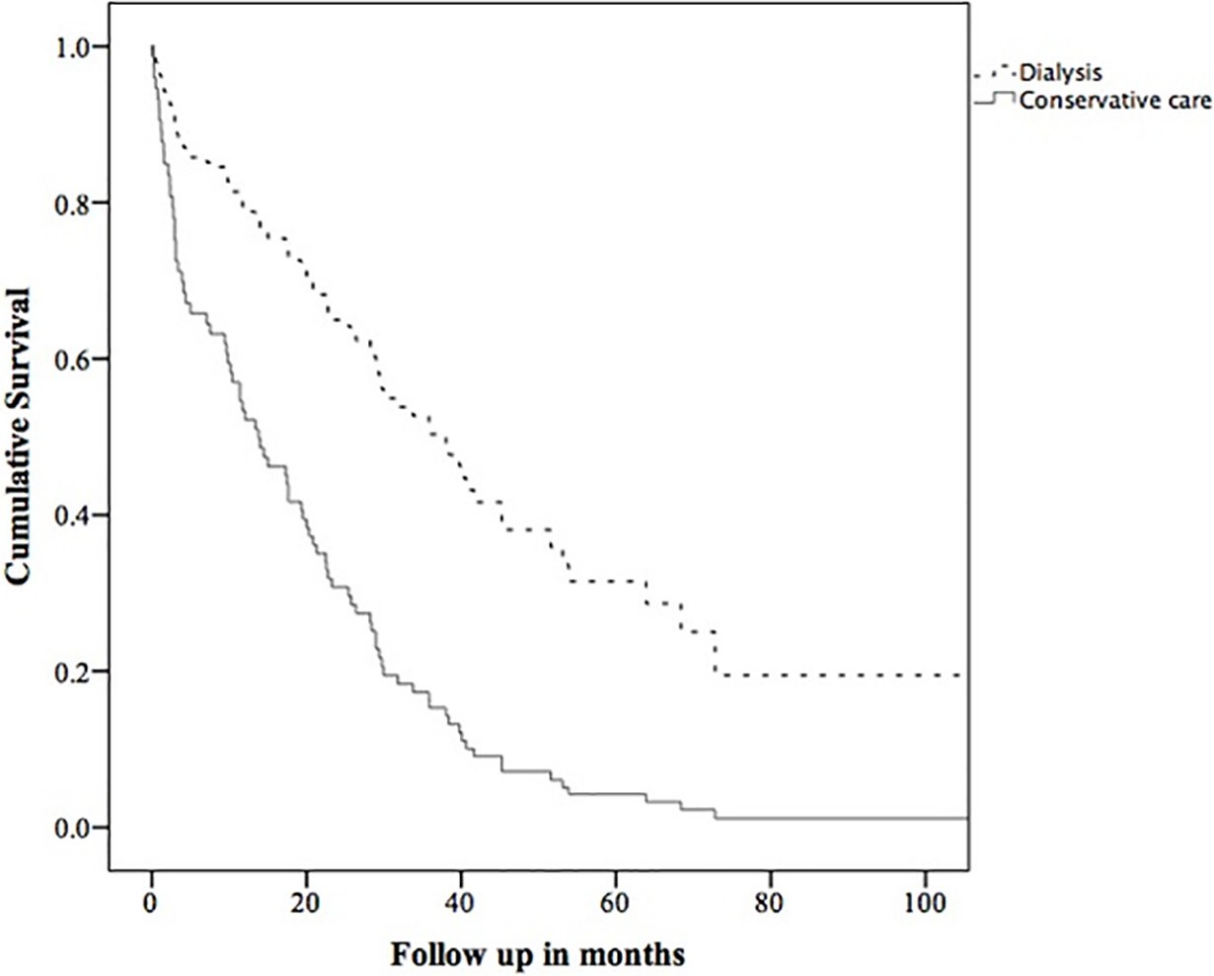
Survival curve comparing dialysis with conservative care from the date of first outpatient eGFR ≤10 mL/min/1.73m^2^. Adjusted for age and peripheral vascular disease.

The survival analysis from first eGFR ≤10 mL/min/1.73m^2^ using a Cox proportional hazard model was adjusted for age, and PVD. The adjusted hazard ratio for death in the dialysis group compared to the conservative care group was 0.36 (0.21–0.62, p = <0.001). An adjusted survival curve comparing groups is shown in Fig 3. In the propensity score matched cohorts, at one year after eGFR first <10 mL/min/1.73m^2^, the OR for mortality in the dialysis group compared to the conservative care group was 0.14 (0.02–0.75). At three years, the OR was 0.15 (0.03–0.87), and at 5 years was 0.45 (0.03–5.84). These are shown in Table 2.

A comparison of annualised rates for hospital visits and procedures is shown in Table 3, including a comparison of haemodialysis versus peritoneal dialysis. Dialysis patients had more outpatient days than conservative care patients (34.3 [IQR, 9.9–92.8] days versus 9.8 [IQR, 5.1–19.6] days, p <0.001), but there was no difference in in-patient days (4.1 [IQR, 1.1–20.2] versus 7.9 [IQR, 0.0–54.3, p = 0.729). As expected, haemodialysis patients had more out patient days than peritoneal dialysis patients. In a survival model comparing dialysis modalities adjusted for baseline eGFR, the HR for death in haemodialysis patients compared to peritoneal dialysis patients was 4.1 (1.23–13.68, p = 0.02).

We analysed outcome in the sub-group of very advanced age, ≥85 years (n = 38 [dialysis = 6, conservative = 32). Here, the HR for death after eGFR first <15mL/min/1.73m^2^ in the dialysis group compared to conservative care was 0.72 (0.25–2.08, p = 0.537). In this age group, the HR for death after eGFR first <10mL/min/1.73m^2^ in the dialysis group compared to conservative care was 0.15 (0.02–1.19, p = 0.073). In this age group, dialysis patients had a statistically significantly greater number of both renal and non-renal invasive procedures. They did not experience a higher burden of outpatient visits from eGFR first <15mL/min/1.73m^2^ but did so after eGFR first <10mL/min/1.73m^2^. They did not demonstrate a higher number of inpatient days from either point.

We also analysed outcome in the sub-group of patients with previous atherosclerotic vascular events (myocardial infarction, coronary revascularisation including bypass, intervention or amputation for PVD, stroke) (n = 100 [dialysis = 67, conservative = 33). Here, the HR for death after eGFR first <15mL/min/1.73m^2^ in the dialysis group compared to conservative care was 0.43 (0.26–0.71, p = 0.001). In this sub-group, the HR for death after eGFR first <10mL/min/1.73m^2^ in the dialysis group compared to conservative care was 0.34 (0.18–0.65, p = 0.001). In this sub-group, dialysis patients had a statistically significantly greater number of both renal and non-renal invasive procedures than the conservative care group from both time points, and both inpatient and outpatient hospital visits from eGFR first <15mL/min/1.73m^2^ but not so from eGFR first <10mL/min/1.73m^2^.

**Fig 4:**
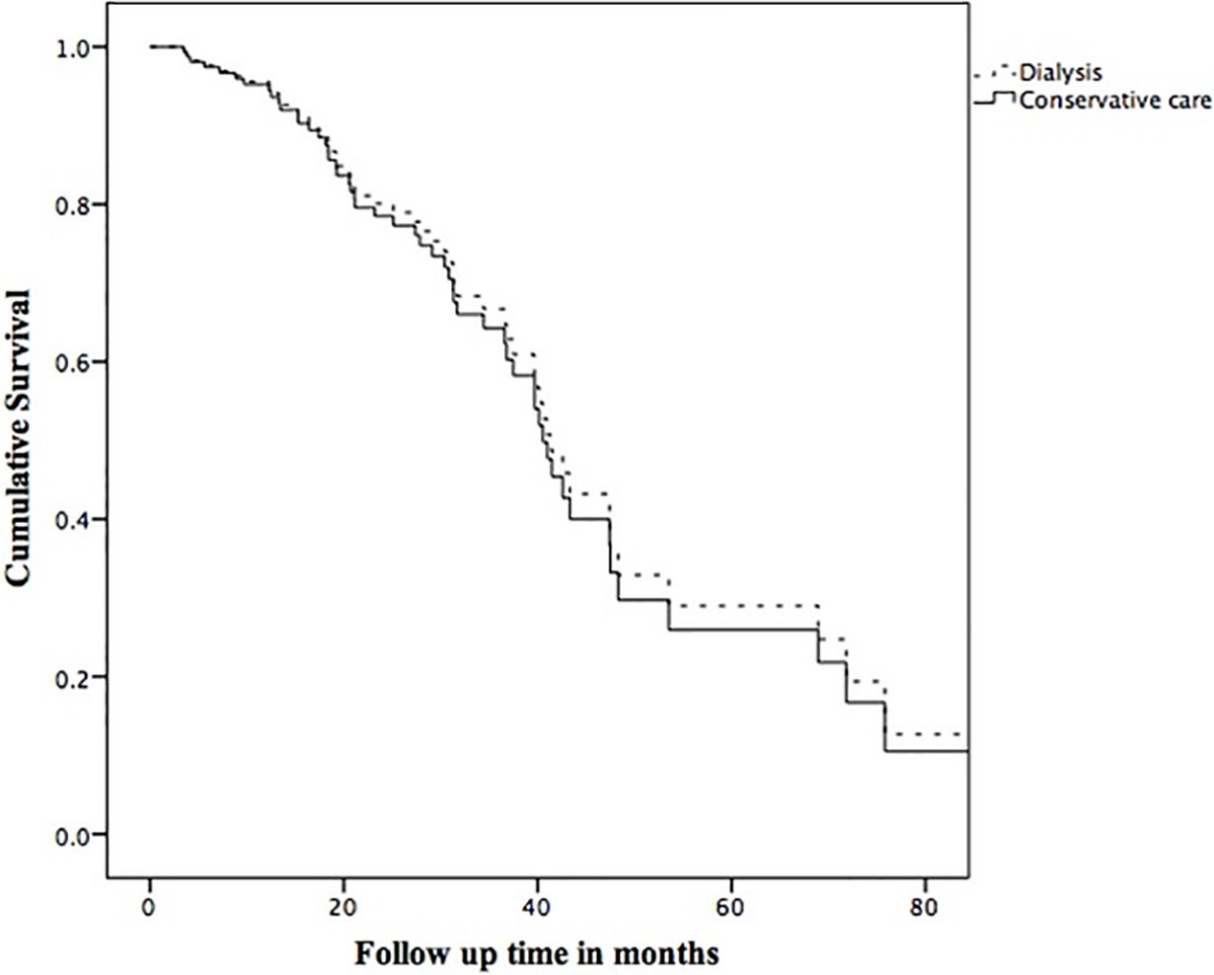
Survival curve comparing dialysis with conservative care during the period that eGFR was between ≤15 mL/min/1.73m^2^ and >10mL/min/1.73m^2^. Adjusted for age, peripheral vascular disease, and living alone.

We also performed survival analysis comparing dialysis versus CC group when their eGFR was between 15 and 10 ml/min/1.73m^2.^ The Kaplan Meier estimates of median survival during this period for dialysis and CC groups were 47.4 months (95% CI = 39.6–55.2) and 39.7 months (95% CI = 29.9–49.4) respectively. The adjusted HR for death in the dialysis compared to the CC group was 0.91 (95% CI = 0.46–1.81), p = 0.80. An adjusted survival curve comparing groups is shown in Fig 4.

We had also anticipated separately comparing dialysis with conservative care in those patients who were excluded from the primary analysis on the basis of medical illnesses, which could be used as a rationale for selecting conservative care. However, of the 24 patients who fulfilled this exclusion criterion (16 heart failure, 7 malignancy, 1 cardiac arrest), only 2 had initiated dialysis during follow up. Of these 2, 1 died within 12 months. 22 of the 24 patients died during a median 39.6 months (IQR 21.6–57.6) follow up.

## Discussion

In this prospectively collected study of older patients with stage 5 CKD who were medically suitable for dialysis, we have shown a statistically significant survival advantage in those patients who chose dialysis. Importantly, this survival benefit was consistent across two statistical methodologies. Both methods also found that the greater survival advantage occurred in the period after eGFR fell below 10mL/min/1.73m^2^. Although is not direct proof of dialysis being the reason for survival advantage, it is very likely that it a major factor.

We have attempted to minimise bias by selecting only patients medically suitable for dialysis, and by using two methods of survival analysis. We have also shown that the clinical phenotypes of the two groups were broadly comparable at both time points selected. We have also show that the rate of change of eGFR in the initial CKD stage 5 period was the same between groups. The limitation remains that patients who chose conservative care were older, more likely to live alone and, of the co-morbidities recorded, had a higher prevalence of PVD. The possibility remains that these factors reflect hidden bias and the extent to which such factors contributed to the difference in outcome remains uncertain. Conversely, although this analysis was performed based on retrospective patient selection, all phenotype data on all patients were collected as part of a detailed prospective observational cohort study. This provides a higher level of phenotype detail than one would typically see in a retrospective study.

The exclusion of patients with pre-existing significantly life limiting illnesses produced an analysis where modality choice was based on patient choice and multi-disciplinary team discussion. This may have also acted to reduce the likelihood of co-morbidities creating bias favouring better outcomes for patients who chose dialysis over conservative care.

Of the 253 patients in our study, 31% (n = 81) chose conservative care, 48% (n = 128) chose dialysis, and 21% (n = 54) did not make a decision regarding RRT during the follow up period when their eGFR was ≤15ml/min/1.73m^2^. In other studies, fewer patients tended to choose conservative care. In a European survey of nephrologist from 2009, conservative care was offered to 10% of patients and an additional 5% chose conservative care when they were offered RRT. This survey included patients of all age groups and was not directed specifically at old people [3]. In a single centre observational study from the United Kingdom evaluating outcomes in old people (≥ 70 years) with ESRD choosing between conservative care and RRT, 14% of patients chose conservative care [4]. The higher percentage of patients choosing conservative care in our study is likely to be due to early multi-disciplinary team review, patient education, and shared decision-making.

It was noted when comparing baseline characteristics of dialysis and CC patients that a statistically greater proportion of CC patients lived alone (40% versus 21%, p = 0.004). A numerically but not statistically greater proportion were widowed (37% versus 24%). This may indicate that social as well as medical factors are key drivers to patient decision making in ESKD.

Late referrals to nephrology services in this age group are associated with much higher mortality. This was 42% in the first year after commencing RTT in one study of 254 patients aged <75 years (77.2%) and ≥75 years (22.8%) [5]. A similar effect has also been described in other age groups. In a national cohort of 2264 dialysis new starters of all ages from the Unites States in the years 1996 and 1997, the first year mortality risk among late referrals was as high as 68% [6] and in another large study of 3014 incident dialysis (peritoneal and haemodialysis) patients from the United States showed that patients who presented late had 36% higher mortality during the first 3 months after starting dialysis compared to patients who presented early [7].

In a retrospective analysis evaluating the outcome of RRT in very old people, Munshi et al showed that the median survival on dialysis for patients ≥75 years was 16 months (95% CI 8–24 months) [8]. We did not specifically evaluate survival from initiation of RRT as the intention was to compare survival with conservative care patients and there is no direct comparator with RRT commencement in conservative care patients.

In a similarly aged patient group (>70 years), a study form the Netherlands showed a survival advantage in the RRT over conservative care group. The median survival was 3.1 (IQR, 1.5–6.9) years versus 1.5 (IQR, 0.7–3) years respectively [9]. This survival advantage was lost in patients age > 80 years and in patients with a Davies comorbidity score of ≥3. However, the survival in that study was calculated form a point when eGFR <20ml/min/1.73m^2^, which is much higher that the eGFR cut off used in our study.

That study did exclude patients who presented as emergency cases but was retrospective and does not appear to have excluded conservative care patients for whom dialysis may have been medically inappropriate. Indeed, we believe our study to be the first significant prospective study in this topic, and the first to exclude conservative care patient for whom dialysis is unlikely to have ever been considered. We believe this to be an important consideration when attempting to undertake as close an approximation to a like-for-like comparison in terms of patient selection. This will better reflect the potential outcome in dialysis versus conservative care for patients in a pre-dialysis setting when undertaking a face to face discussion with them about whether they want dialysis.

Most studies described above have shown a survival advantage with dialysis compared to conservative care. However, there is conflicting information from recent meta-analysis which showed that there is not much difference in survival between dialysis and CC groups. Here, the 1 year survival for undifferentiated dialysis versus conservative care in old people was 73% (95%CI, 66.3–79.7%) and 71% (95%CI, 63.3–78%) respectively [10].

We have reported report significantly greater frequency of outpatient and inpatient follow up visits in the “dialysis group” patients compared to those on conservative care. Whilst the majority refer to preparation for dialysis, dialysis itself, and complications of dialysis, it is also likely that dialysis patients received more aggressive care in general. Timely treatment of even apparently minor ailments can prevent severe complications in patients with such advanced CKD.

## Conclusion

Our prospective observational study favours the standpoint that dialysis does confer a survival advantage in older people aged ≥75 years with end stage kidney disease. The survival in our study was assessed from two crucial points in a patient’s journey towards end stage kidney disease. The survival advantage seen in our study provides valuable information for decision making in the age group. Our study had a high uptake into conservative care compared to other studies.

## Limitations

The limitations to our study are that we did not undertake measurements of quality of life to compare this aspect of outcome between groups. Also, as with all studies that are not randomised, the possibility of bias remains.

## References

[1] Caskey F, Castledine C, Dawnay A, Farrington K, Fogarty D, Fraser S, et al UK Renal Registry. 18th Annual Report of the Renal Association. Nephron. 2016; 132: 1–366.

[2] Lamping DL, Constantinovici N, Roderick P, Normand C, Henderson L, Harris S, et al Clinical outcomes, quality of life, and costs in the North Thames Dialysis Study of elderly people on dialysis: a prospective cohort study. Lancet. 2000; 356(9241): 1543–1550. 10.1016/S0140-6736(00)03123-8 11075766

[3] van de Luijtgaarden MW, Noordzij M, van Biesen W, Couchoud C, Cancarini G, Bos WJ, et al Conservative care in Europe—Nephrologists’ experience with the decision not to start renal replacement therapy. Nephrol Dial Transplant. 2013; 28(10): 2604–2612. 10.1093/ndt/gft287 24013682

[4] Carson C, Juszczak M, Davenport A, Burns A. Is maximum conservative management an equivalent treatment option to dialysis for elderly patients with significant comorbid disease? Clin J Am Soc Nephrol. 2009; 4(10): 1611–1619. 10.2215/CJN.00510109 19808244 PMC2758251

[5] Schwenger V, Morath C, Hofmann A, Hoffmann O, Zeier M, Ritz E. Late referral—A major cause of poor outcome in the very elderly dialysis patient. Nephrol Dial Transplant. 2006; 21(4): 962–967. 10.1093/ndt/gfk030 16396974

[6] Stack AG. Impact of timing of nephrology referral and pre-ESRD care on mortality risk among new ESRD patients in the United States. Am J Kidney Dis. 2003; 41(2): 310–318. 10.1053/ajkd.2003.50038 12552491

[7] Winkelmayer WC. A Propensity Analysis of Late Versus Early Nephrologist Referral and Mortality on Dialysis. J Am Soc Nephrol. 2003; 14(2): 486–492. 12538751 10.1097/01.asn.0000046047.66958.c3

[8] Munshi SK, Vijayakumar N, Taub NA, Bhullar H, Lo TC, Warwick G. Outcome of renal replacement therapy in the very elderly. Nephrol Dial Transplant. 2001; 16(1): 128–133. 11209006 10.1093/ndt/16.1.128

[9] Verberne WR, Geers ABMT, Jellema WT, Vincent HH, Van Delden JJM, Jan W, Bos W. Comparative Survival among Older Adults with Advanced Kidney Disease Managed Conservatively Versus with Dialysis. Clin J Am Soc Nephrol. 2016; 18: 1–8. 10.2215/CJN.07510715 PMC4822664 26988748

[10] Foote C, Kotwal S, Gallagher M, Cass A, Brown M, Jardine M. Survival outcomes of supportive care versus dialysis therapies for elderly patients with end-stage kidney disease: A systematic review and meta-analysis. Nephrology. 2016; 21(3): 241–253. 10.1111/nep.12586 26265214

